# Inactivation of apaziquone by haematuria: implications for the design of phase III clinical trials against non-muscle invasive bladder cancer

**DOI:** 10.1007/s00280-019-03812-7

**Published:** 2019-03-13

**Authors:** Roger M. Phillips, Paul M. Loadman, Guru Reddy

**Affiliations:** 1 Department of Pharmacy, School of Applied Sciences, University of Huddersfield, Huddersfield, HD1 3DH UK; 2 Institute of Cancer Therapeutics, University of Bradford, Bradford, BD7 1DP UK; 3 Spectrum Pharmaceuticals Inc, Irvine, CA USA

**Keywords:** Apaziquone, Non-muscle invasive bladder cancer, Haematuria

## Abstract

**Purpose:**

Despite positive responses in phase II clinical trials, the bioreductive prodrug apaziquone failed to achieve statistically significant activity in non-muscle invasive bladder cancer in phase III trials. Apaziquone was administered shortly after transurethral resection and here we test the hypothesis that haematuria inactivates apaziquone.

**Methods:**

HPLC analysis was used to determine the ability of human whole blood to metabolise apaziquone ex vivo. An in vitro model of haematuria was developed and the response of RT112 and EJ138 cells following a 1-h exposure to apaziquone was determined in the presence of urine plus or minus whole blood or lysed whole blood.

**Results:**

HPLC analysis demonstrated that apaziquone is metabolised by human whole blood with a half-life of 78.6 ± 23.0 min. As a model for haematuria, incubation of cells in media containing up to 75% buffered (pH 7.4) urine and 25% whole blood was not toxic to cells for a 1-h exposure period. Whole blood (5% v/v) significantly (*p* < 0.01) reduced the potency of apaziquone in this experimental model. Lysed whole blood also significantly (*p* < 0.05) reduced cell growth, although higher concentrations were required to achieve an effect (15% v/v).

**Conclusions:**

The results of this study demonstrate that haematuria can reduce the potency of apaziquone in this experimental model. These findings impact upon the design of further phase III clinical trials and strongly suggest that apaziquone should not be administered immediately after transurethral resection of non-muscle invasive bladder cancer when haematuria is common.

## Introduction

Apaziquone, originally known as EO9 (3-hydroxy-5-aziridinyl-1- methyl-2-(1H-indole-4,7-dione)prop-β-en-α-ol), has had a long and chequered history culminating in its clinical evaluation against non-muscle invasive bladder cancer [1, 2]. Whilst its pharmacokinetic properties are undesirable for systemic administration, they are paradoxically advantageous in the treatment of non-muscle invasive bladder cancer (NMIBC) where intravesical administration ensures adequate delivery to the tumour site and any drug leaching out into the blood stream is rapidly removed leading to low risk of systemic side effects. Apaziquone is a bioreductive prodrug that requires enzymatic activation to DNA damaging species [2] and following the demonstration that NMIBC possesses the appropriate enzymology to activate apaziquone [3, 4], significant ablative activity against NMIBC marker lesions was reported in phase I and II clinical trials [5, 6]. The level of activity observed in marker lesion studies was higher than that reported for other chemotherapy drugs and immune response modifiers [7] and 2-year response rates demonstrated that long-term responses were good in comparison to other ablative studies [8, 9]. The results of two phase III clinical trials were, however, disappointing, and when analysed individually, both studies failed to reach statistical significance for the primary end point which was the 2 year recurrence rate [10].

To try and explain the stark differences in outcomes between the phase III trial and marker lesion studies, we focus in this study on key differences in the design of these clinical trials. The major difference between the two sets of studies was that a single dose of apaziquone was administered intravesically within 24 h of transurethral resection of bladder tumours (TURBT) in phase III studies, whereas apaziquone was administered weekly for 6 weeks starting 2 weeks after TURBT in the marker lesion studies. There is therefore a difference in the timing of drug administration relative to TURBT and a reduction in the cumulative dose given to patients in the phase III study. Haematuria is common following surgical resection of tumours and, in 2002, we demonstrated that apaziquone is rapidly metabolised by murine whole blood [11]. Whilst the context of these studies focused on the design of second-generation analogues of apaziquone that had better pharmacokinetic properties, this observation has direct relevance to the clinical problem outlined above. Administration of apaziquone immediately after surgery when haematuria is common could affect the potency and therefore efficacy of apaziquone. Post hoc analysis of the data generated in the phase III trial did in fact reveal that a subset of patients who received apaziquone within 30 min of TURBT had no difference in response rate compared to the placebo arm [10]. In contrast, patients who received apaziquone between 30 and 90 min after TURBT demonstrated a highly significant response to apaziquone in terms of 2-year recurrence rates. Our hypothesis therefore is that haematuria immediately after surgery causes a reduction in the efficacy of apaziquone when administered immediately after TURBT. In this study, experimental evidence is provided demonstrating that apaziquone is metabolised by human blood and this reduces the potency of apaziquone in an in vitro model of haematuria. These findings have implications for the design of future phase III clinical trials and these are discussed within.

## Materials and methods

### Metabolism of apaziquone by human blood

The metabolism of apaziquone by human whole blood was performed as described elsewhere [11]. Following informed consent, blood samples were obtained from healthy volunteers, collected in EDTA tubes [BD vacutainer K2E (EDTA)] and either used as ‘whole blood’ or plasma (obtained by centrifuging blood at 3000*g* for 5 min). Whole blood or plasma samples were spiked with apaziquone (20 µM) and incubated at 37 °C for various times. Apaziquone was extracted from samples by the addition of ice-cold acetonitrile with a solvent/sample ratio of 2:1 and centrifuged at 7000*g* for 5 min to remove precipitated proteins. Samples were analysed by reverse phase HPLC [12] using a LiChrosorb RP-18 column and a Waters 996 Photodiode Array detector. The mobile phase was phosphate buffer (10 mM, pH 7.0)/methanol (57/43) and the flow rate was 1.2 ml/min. Identical studies were conducted using phosphate-buffered saline (PBS at pH 7.4). PBS spiked with 20 µM apaziquone (*t* = 0) was used as the 100% control and recovery from biological samples was expressed as a percentage of the PBS control.

### Cell lines and chemosensitivity studies

RT112 and EJ138 human bladder carcinoma cell lines were obtained from ECACC and routinely maintained as monolayer cultures in RPMI1640/DMEM (50:50 mix) containing foetal calf serum (10% v/v) and l-glutamine (2 mM). Purified, unformulated apaziquone was obtained from Spectrum Pharmaceuticals Inc. and stock solutions at 100 mM were prepared in DMSO, aliquoted and stored at − 20 °C. For chemosensitivity studies performed under standard cell culture conditions, cells were plated into 96-well plates at 2 × 10^3^ cells per well and incubated overnight to adhere. The following day, cell culture media were removed and replaced with media containing a range of apaziquone concentrations (10–0.196 µM using a twofold serial dilution strategy). Cells were exposed to apaziquone for 1 h (to mimic the 1-h instillation protocol used in the clinic), following which they were washed three times with PBS before the addition of 200 µl of media to each well. Cells were subsequently incubated at 37 °C for 96 h before growth inhibition determination using the MTT assay as described elsewhere [12]. Briefly, culture media were removed from each well and replaced with 200 µl of fresh media prior to the addition of 20 µl of MTT (5 mg/ml). Following a 4-h incubation at 37 °C, media were removed and formazan crystals dissolved in 150 µl of DMSO per well. The absorbance of the resulting solution was determined spectrophotometrically at 540 nm and cell growth was determined as the true absorbance of treated cells divided by the true absorbance of control cells and expressed as a percentage. Three independent experiments were performed and the results are presented as the mean IC_50_ values ± standard deviation.

Apaziquone is known to be active against both aerobic and hypoxic cells [13], but all studies were conducted against aerobic cells for the following reasons. First, the phase III studies were conducted in patients that had all tumours surgically removed and therefore there were no solid masses where hypoxia could exist. Second, the vehicle used in the instillation was well oxygenated (in equilibrium with atmospheric conditions) and the administration of 40 ml directly into the bladder would be expected to create local conditions that would be aerobic. Based on the above considerations, it would be unlikely that conditions in the bladder would be conducive to the hypoxia activation of apaziquone and this was therefore not evaluated.

### Chemosensitivity studies in the presence of urine and whole blood

To determine the amount of blood and urine that cells in culture will tolerate, cells were exposed to varying amounts of urine or blood for 1 h and cell survival was determined 96 h later using the MTT assay as described above. Following informed consent, blood samples were obtained from healthy volunteers and placed into EDTA tubes. For studies using whole blood, samples were initially diluted 1:1 in cell culture media followed for subsequent twofold serial dilution in media to generate a range of blood dilutions (50–0.098% v/v). Cell culture plates were set up as described above and following a 1-h exposure to media containing whole blood at different concentrations, cells were washed, culture media added (200 µl/well) and incubated for a further 96 h before percentage growth inhibition was determined using the MTT assay as described above. In addition, as haemolysis is commonly observed in haematuria, similar studies were conducted using lysed whole blood (obtained by repeated cycles of rapid freezing in liquid nitrogen and thawing at 37 °C).

Mid-flow urine samples were obtained from healthy volunteers and used immediately. Urine samples were either applied directly to cells or pH adjusted to 7.4 using NaOH prior to addition to cells. Unbuffered or pH-adjusted urine was diluted in culture media to give a range of urine concentrations and these were applied to cell cultures as described above. Following a 1-h incubation, cells were washed and culture media added (200 µl/well). Following a further 96-h incubation at 37 °C, percentage cell growth was determined using the MTT assay as described above.

## Results

### Metabolism of apaziquone by whole blood and plasma

**Fig. 1:**
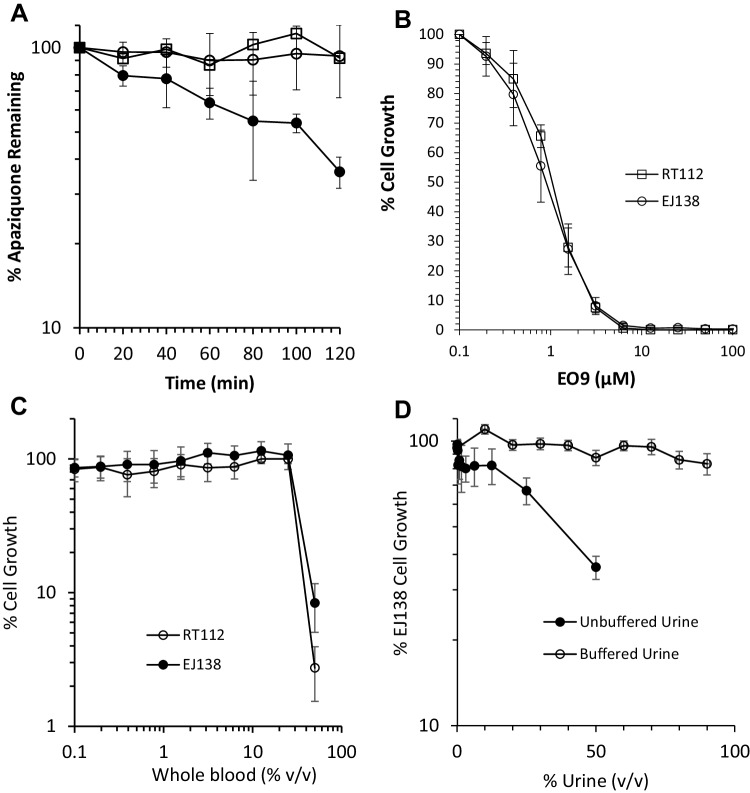
Metabolism of apaziquone by human blood and establishment of the haematuria model. **a** Time-dependent metabolism of apaziquone following incubation at 37 °C in whole blood (filled circle), plasma (unfilled circle) and PBS (unfilled square). Each value represents the mean ± standard deviation for three independent experiments. The response of EJ138 and RT112 cells following a 1-h exposure to EO9 is presented in **b**. Values represent the mean ± standard deviation for three independent experiments. The response of cells to whole blood and urine are presented in **c** and **d**, respectively. Exposure times were 1 h and each result represents the mean ± standard deviation for eight replicates

The metabolism of apaziquone in human whole blood, human plasma and PBS is presented in Fig. 1a. Whilst apaziquone is stable in PBS and plasma over a 2-h period, levels of apaziquone decrease significantly in a time-dependent manner (Fig. 1a). No metabolites (specifically the inactive products EO5A and EO9-Cl [14]) were observed on chromatograms (data not shown) suggesting that any active metabolites of apaziquone are irreversibly bound to cellular components of whole blood and remain within the protein precipitate.

### Response of EJ138 and RT112 cells to apaziquone

The response of cell lines following a 1-h exposure to apaziquone are presented in Fig. 1b. Both cell lines are sensitive to apaziquone with IC_50_ values of 1.11 ± 0.09 and 0.915 ± 0.291 µM for RT112 and EJ138 cells, respectively. In both cell lines, a 1-h exposure to apaziquone at 5 µM induces a 95% growth inhibition, so this concentration was chosen for subsequent experiments to determine the effect of blood and urine on the activity of apaziquone.

### Response of EJ138 and RT112 cells to urine and whole blood

The effect of whole blood and urine on the proliferation of cells is presented in Fig. 1c, d, respectively. The addition of whole blood onto cells for a period of 1 h has no effect on cell survival up to 25% v/v. At 50% v/v, however, substantial cell killing occurred along with problems with clotting in the wells. For subsequent experiments, therefore, the highest concentration of whole blood used was 25% v/v. Whilst difficult to establish the clinical relevance of this concentration of whole blood in these experiments, it is known that haematuria following TURBT is both common and can vary between mild to gross haematuria that requires further medical intervention [15]. Exposure of cells to unbuffered urine (in the experiment in Fig. 1d, the pH of the urine was 5.93) led to a dose-dependent reduction in cell growth. Cells were however able to tolerate up to 90% urine once pH had been adjusted to pH 7.4. For subsequent experiments, pH-adjusted urine (pH 7.4) at 75% (v/v) was used.

In summary, the results presented in Fig. 1b–d define the parameters within which subsequent experiments were performed. These include (i) the concentration of apaziquone (5 µM) that induces > 95% cell growth inhibition, (ii) an upper limit for the amount of whole blood that can be used (25% v/v) without affecting cell growth and (iii) the amount of buffered urine that can be used without causing significant inhibition of cell growth (75% v/v).

### Response of EJ138 and RT112 cells to apaziquone in buffered urine and whole blood

**Fig. 2:**
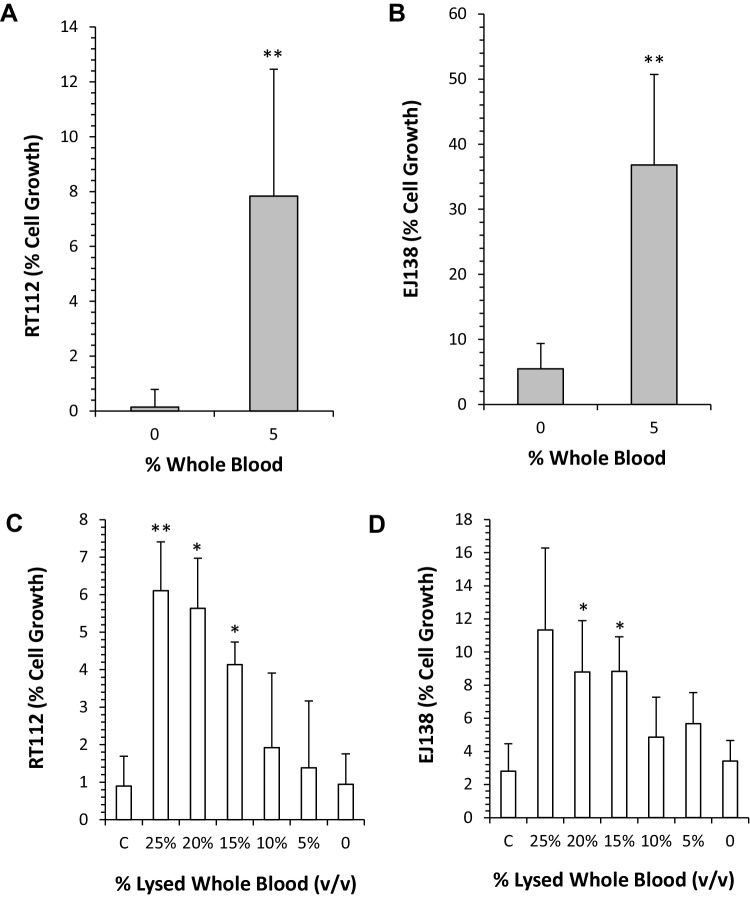
Response of RT112 and EJ138 cells to EO9 in the presence and absence of whole blood and lysed whole blood. **a, b** Response of RT112 and EJ138, respectively, to a 1-h exposure to apaziquone (5 µM) in buffered urine (75% v/v) without (0) or with whole blood (5% v/v). Following drug exposure, cell growth was determined 96 h later using the MTT assay. **c, d** RT112 and EJ138 treated with apaziquone (5 µM), urine (75%) and various concentrations of lysed whole blood. Exposure to experimental conditions was for 1 h, followed by a 96 h recovery period prior to the assessment of cell growth using the MTT assay. Control experiments (**c**) used media plus apaziquone only (no urine or lysed whole blood). Statistical analysis was performed using a paired *t* test comparing the effects of each treatment with that of controls (** and * denote statistical significance at *p* < 0.01 and *p* < 0.05, respectively)

The results presented in Fig. 2a, b clearly demonstrate that the inclusion of whole blood (5%) and buffered urine (75%) significantly (*p* < 0.01) reduces the potency of apaziquone. Whilst apaziquone induces a > 95% cell killing in both RT112 and EJ138 cells in the absence of whole blood, the inclusion of whole blood (5% v/v) significantly increases cell survival in RT112 (*p* = 0.0029) and EJ138 (*p* = 0.0004) cells. The effect of buffered urine on the activity of apaziquone is negligible, as cell growth inhibition in the positive controls is similar to cell growth inhibition in media (compare Fig. 2a, b with Fig. 1b). The results obtained using lysed whole blood are presented in Fig. 2c, d. In both cell lines, a small but statistically significant (*p* < 0.05) reduction in the potency of apaziquone was observed when lysed whole blood (at concentrations of 15% and above) was added to buffered urine. This effect was dose dependent with respect to the amount of lysed whole blood included in the assay. In contrast to the whole blood experiments where highly significant reductions in activity were observed at 5% whole blood, the effect of lysed whole blood on the activity of apaziquone was lower.

## Discussion

The main reason for the change in the protocols between apaziquone phase III and marker lesion studies was to comply with current guidance for intravesical chemotherapy issued by the European Association of Urology (EAU) and American Urological Association (AUA). A series of studies indicate that a single instillation of chemotherapy immediately after resection reduces the risk of recurrence [16–19] with the most benefit seen with mitomycin C and epirubicin. The EAU guidelines therefore recommend immediate intravesical instillation of chemotherapy after surgical resection of low to intermediate risk NMIBC. The AUA guidelines differ slightly in that intravesical administration of chemotherapy (mitomycin C or epirubicin) should be considered within 24 h of surgical resection for low to intermediate risk NMIBC patients. Whilst there is strong clinical evidence for the immediate intravesical administration of mitomycin C or epirubicin after TURBT, the results of this study suggest that the same principles and guidance may not be applicable to apaziquone.

In contrast to the structurally related mitomycin C which is not metabolised by murine whole blood, our previous studies have demonstrated that apaziquone is rapidly metabolised by murine whole blood [11]. In this study we demonstrate that apaziquone is also metabolised by human blood (Fig. 1a), albeit at a reduced rate compared to murine blood. The fact that apaziquone is stable in both PBS and plasma suggests that chemical instability and protein binding are not responsible for the loss of apaziquone. Furthermore, the fact that no metabolites were detected using HPLC supports the argument that apaziquone is being metabolised to products that are irreversibly bound to cellular macromolecules that cannot be recovered using acetonitrile extraction techniques.

Apaziquone is a prodrug that requires enzymatic activation to generate cytotoxic intermediates with one and two electron oxidoreductases playing a prominent role in drug activation [2]. The identification of enzymes capable of metabolising apaziquone in human blood has not been elucidated in this study, but it is important to recognise that the RBC component of whole blood is not just concerned with the transport and release of oxygen but it has other metabolic functions that impact upon the pharmacokinetic and pharmacodynamic property of drugs including anti-cancer agents [20, 21]. These metabolic functions extend to redox reactions, nitric oxide metabolism and glutathione metabolism [22, 23], all of which can affect the pharmacology of anti-cancer drugs. Furthermore, red blood cells are known to contain high levels of NADH cytochrome b5 reductase (CYB5R), an enzyme that has been implicated in the bioreductive activation of quinone-based drugs including mitomycin C [24, 25]. A direct role for CYB5R in the activation of apaziquone has not however been established and further studies are required to fully understand the detailed metabolism of apaziquone by human whole blood. The impact of this study however resides in the development of an in vitro model of haematuria and the demonstration that the efficacy of apaziquone is reduced under experimental conditions that mimic haematuria. This study suggests that the administration of apaziquone immediately after TURBT when haematuria is common could reduce the efficacy of apaziquone. Whilst the level of haematuria was not reported in phase III clinical trials, the presence of whole blood or lysed whole blood in the bladder immediately (less than 30 min) after TURBT is a common observation and therefore a potential contributing factor that could explain the reduced efficacy of apaziquone observed in phase III clinical trials.

With regard to the continued development of apaziquone, a further phase III clinical trial is required to address the post hoc analysis of the results and subsequent hypotheses generated during the previous phase III studies (FDA briefing document). Based on the results of this study, immediate instillation of apaziquone should not be performed with a preference for instillation 60 ± 30 min after surgery when haematuria is reduced or non-existent. Another potential way of circumventing the effects of haematuria is to increase the dose administered. A dose escalation was performed in a clinical pilot study [5] and 4 mg/40 ml was selected as the dose that induced no systemic or local side effects. This dose was used for all subsequent clinical studies including the phase III clinical trials. A dose of 8 mg/40 ml induced some local toxicities (grade 2/3 dysuria and haematuria), but was also well tolerated.

In conclusion, the results of this study have demonstrated that haematuria can reduce the efficacy of apaziquone in experimental models in vitro and this is a potential explanation for the poor efficacy of apaziquone when administered within 30 min of TURBT. These results were used to inform the design of the current active phase III clinical trial (NCT03224182) where apaziquone is administered intravesically 60 ± 30 min after TURBT at a dose of 8 mg/40 ml.
